# Fabrication and Characterization of Single-Crystal Diamond Membranes for Quantum Photonics with Tunable Microcavities

**DOI:** 10.3390/mi11121080

**Published:** 2020-12-04

**Authors:** Julia Heupel, Maximilian Pallmann, Jonathan Körber, Rolf Merz, Michael Kopnarski, Rainer Stöhr, Johann Peter Reithmaier, David Hunger, Cyril Popov

**Affiliations:** 1 Institute of Nanostructure Technologies and Analytics (INA), Center for Interdisciplinary Nanostructure Science and Technology (CINSaT), University of Kassel, Heinrich-Plett-Straße 40, 34132 Kassel, Germany; j.heupel@ina.uni-kassel.de (J.H.); reithmaier@ina.uni-kassel.de (J.P.R.); 2 Physikalisches Institut, Karlsruher Institute für Technologie (KIT), Wolfgang-Gaede-Str.1, 76131 Karlsruhe, Germany; maximilian.pallmann@kit.edu (M.P.); jonathan.koerber@student.kit.edu (J.K.); david.hunger@kit.edu (D.H.); 3 Institut für Oberflächen- und Schichtanalytik (IFOS), University of Kaiserslautern, Trippstadter Str. 120, 67663 Kaiserslautern, Germany; merz@ifos.uni-kl.de (R.M.); kopnarski@ifos.uni-kl.de (M.K.); 4 Physikalisches Institut, University of Stuttgart, Pfaffenwaldring 57, 70569 Stuttgart, Germany; rainer.stoehr@pi3.uni-stuttgart.de; 5 Institut für QuantenMaterialien und Technologien (IQMT), Karlsruher Institut für Technologie, Hermann-von-Helmholtz-Platz 1, 76344 Eggenstein-Leopoldshafen, Germany

**Keywords:** single-crystal diamond, membranes, fiber-based microcavity, nanophotonics, micromasking, roughness reduction

## Abstract

The development of quantum technologies is one of the big challenges in modern research. A crucial component for many applications is an efficient, coherent spin–photon interface, and coupling single-color centers in thin diamond membranes to a microcavity is a promising approach. To structure such micrometer thin single-crystal diamond (SCD) membranes with a good quality, it is important to minimize defects originating from polishing or etching procedures. Here, we report on the fabrication of SCD membranes, with various diameters, exhibiting a low surface roughness down to 0.4 nm on a small area scale, by etching through a diamond bulk mask with angled holes. A significant reduction in pits induced by micromasking and polishing damages was accomplished by the application of alternating Ar/Cl_2_ + O_2_ dry etching steps. By a variation of etching parameters regarding the Ar/Cl_2_ step, an enhanced planarization of the surface was obtained, in particular, for surfaces with a higher initial surface roughness of several nanometers. Furthermore, we present the successful bonding of an SCD membrane via van der Waals forces on a cavity mirror and perform finesse measurements which yielded values between 500 and 5000, depending on the position and hence on the membrane thickness. Our results are promising for, e.g., an efficient spin–photon interface.

## 1. Introduction

In recent years, single-crystal diamond (SCD) membranes have emerged as a highly promising platform for various application fields including photonic integrated devices [1,2,3], radiation and pressure sensing [4,5] and nanoelectronics [6,7] due to diamond’s exceptional electrical [8,9,10] and optical properties [11,12]. Especially for envisioned applications in quantum sensing [13,14] and in quantum information technologies (QITs), such as quantum memories [15,16] and quantum communication [17,18,19], SCD gained, as a host material, an ever-increasing scientific interest based on remarkable properties of different optically active point defects in its crystal lattice—the so-called color centers [20]. A long coherence time for these defect related electronic states is facilitated by the wide-bandgap material (≈5.5 eV for bulk diamond), since diamond crystals are relatively free of background nuclear spins and feature a low electron concentration as well as low phonon scattering rate [21,22]. These color centers, such as the nitrogen-vacancy (NV) center [23,24], the silicon-vacancy (SiV) center [25,26] or the germanium-vacancy center (GeV) [27,28], exhibit a high photostability at room temperature, polarizability and allow a practicable control of coherent single spins, hence serving as single-photon emitters. In particular, the prominent NV color center presents a promising candidate for quantum memory bits due to its long-lived and well-defined spin quantum states [29].

To yield an efficient outcoupling from the zero-phonon line (ZPL) of the color centers and improve the photon collection efficiency, various light-confining architectures can be used, such as Fabry–Pérot microcavities [30,31], nanopillars [32,33,34] or photonic crystal cavities [35]. In particular, cavities with small mode volumes and high quality factors can lead to selective and strong Purcell enhancement of the ZPL emission, an important ingredient for coherent single-photon sources, fast spin readout and an efficient creation of spin–photon entanglement. However, nanofabrication produces near-surface damage, and in particular for the NV center, it was found that this leads to a substantial degradation of the optical coherence properties. The most promising approach is to use minimally processed diamond membranes with a thickness of a few micrometers where color centers remain far away from surfaces, which are introduced into open-access microcavities by bonding to one of the cavity mirrors [25,26,36]. Such microcavities allow for spatial and spectral tunability in order to controllably match the geometrical and spectral overlap of a cavity mode with a selected color center. Furthermore, fiber-based microcavities allow for a direct outcoupling of photons in a single-mode fiber [24,37,38]. The central challenge for this approach is that additional losses introduced by the diamond can limit the efficiency of the system, and the surface roughness of the diamond membrane is the most crucial parameter. To minimize losses due to scattering and maintain a high finesse of the cavity, the surface roughness should be as low as possible, preferably in the sub-nanometer regime on a small area [39]. Our simulations of a hybrid membrane-cavity system suggest that a root mean square (rms) roughness of <0.5 nm is necessary for an effective Purcell enhancement. However, different surface defects such as pits, grooves and dislocations originating from polishing [40] and further processing of diamond substrates, e.g., structuring by dry etching procedures, can pose a challenge to reach a low surface roughness and hence fabricate high quality SCD membranes [41]. Mechanical polishing, commonly in the form of scaife polishing to remove diamond material, can introduce polishing damage extending into the bulk region under the processed surface from several hundred nanometers up to 10 μm in depth [42,43]. Besides polishing damage, another significant source of defects causing a higher surface roughening is the micromasking effect. Due to particles on the surface, e.g., originating from the polishing procedure or sputtered material during the reactive ion etching (RIE) process, holes or etch pits can be formed at the surface [24].

In the current work, we present an approach for fabrication of thin SCD membranes with a thickness in the range of 2–5 µm, utilizing a diamond bulk mask with angled holes to withstand long etch procedures. Besides a systematic examination by varying the etching parameters to reduce micromasking and the surface roughness, the importance of a thorough cleaning procedure before structuring is highlighted. As a second step, we report on the integration and characterization of the optimized membranes in a fiber-based Fabry–Pérot microcavity. We present a bonding technique for large diamond samples that utilizes van der Waals attraction between two surfaces. We characterize the microcavity mode structure and loss in the presence of the incorporated diamond membrane.

## 2. Materials and Methods

### 2.1. Fabrication of Single-Crystal Diamond (SCD) Membranes

**Figure 1:**
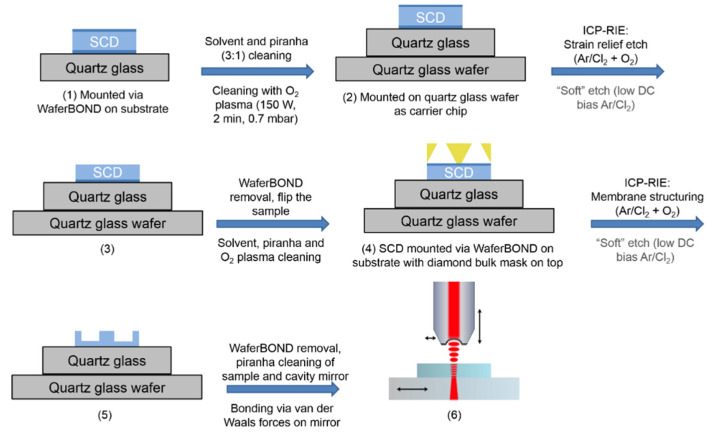
Fabrication process of single-crystal diamond (SCD) membranes and characterization in a fiber-based microcavity: in dark blue the surface with a higher roughness is indicated, while in yellow the bulk diamond mask with angled holes is depicted. Since the “soft” etch was applied only for samples with a higher roughness (>1 nm on a 4 × 4 µm^2^ area), it is highlighted in grey.

For the structuring of SCD membranes, two kinds of chemical vapor deposited (CVD) diamond samples with distinctions in purity were used: general grade SCD (Cornes Technologies, San Jose, CA, USA) and electronic grade SCD (Element Six, London, UK, [N_s_]^0^ < 5 ppb and [B] < 1 ppb) according to manufacturers’ specifications. For the electronic grade samples, the cutting and mechanical polishing processes were executed by Delaware Diamond Knives (DDK, Wilmington, DE, USA) to obtain lateral dimensions of 2 × 2 mm^2^ and a thickness in the range of 30–40 μm. Regarding the general grade samples, the cutting was carried out with a green laser system and the polishing via a diamond lapping system at Cornes Technologies, USA. The overall fabrication process is depicted in detail in Figure 1. For better handling, these samples were mounted via an adhesive (WaferBOND^®^ HT-10.11, Brewer Science, Rolla, MO, USA) on a larger quartz glass substrate (1 × 1.2 cm^2^) (Figure 1 (1)), which was cleaned beforehand with acetone, isopropanol and piranha solution (3:1, H_2_SO_4_:H_2_O_2_) to remove any contaminations. For the bonding, each SCD sample was placed via vacuum tweezers on a small amount of adhesive, dropped via a needle onto the substrate. To bake the adhesive layer, the sample was placed on a hot plate at 180 °C for serval minutes. Afterwards, the SCD plates fixed on the substrate were cleaned again with acetone, isopropanol and finally in piranha solution (3:1) for 45 min up to 1 h. After rinsing of the samples in ultrapure water for several minutes, the samples were dried with nitrogen flow. Besides the control with an optical microscope, whether the samples featured a significant amount of particles or contaminations was examined by atomic force microscopy (AFM, DualScope 95, DME). If detected, the cleaning procedure was repeated. Additionally, the samples were cleansed and O-terminated in an oxygen plasma asher (TePla 200-G, 150 W, 0.7 mbar, 2 min). 

After the cleaning procedure, a strain relief etch [44,45] of several microns from the likewise polished backside of the sample was executed in an inductively coupled plasma reactive ion etching (ICP-RIE) system (Oxford Plasmalab 100). The sample was fixed on a quartz glass wafer as carrier chip for transfer into the reaction chamber (Figure 1 (2)) and subjected to etching in order to remove potentially damaged material and stress in the layer (Figure 1 (3)). The etching recipe involved alternating Ar/Cl_2_ and O_2_ steps, as derived from the work of Ruf et al. [24]. The ICP-RIE process parameters were as following for the Ar/Cl_2_ step: RF power = 200 W, ICP power = 500 W, *p* = 7 mTorr, Ar flow = 10 sccm, Cl_2_ flow = 20 sccm, T_sub_ = 30 °C, and for the O_2_ step: RF power = 90 W, ICP power = 1100 W, *p* = 7 mTorr, O_2_ flow = 50 sccm, T_sub_ = 20 °C. For a further reduction in the surface roughness, especially for samples exhibiting roughness values above 1 nm on a small area scale, a “soft” etch with Ar/Cl_2_ was applied. By decreasing the RF power to 40 W and the ICP power to 200 W, a low DC bias value of 40 V was achieved. 

For structuring of membranes, the adhesive layer was removed by immersing the samples into a WaferBOND^®^ remover bath for several hours until the SCD plate detached from the substrate. Afterwards, the sample was flipped and the gluing/cleaning procedure as explained in the previous paragraph was repeated. To withstand the long dry etching procedures in order to reach a final thickness of a few micrometers, a bulk diamond mask (Medidia GmbH, Idar-Oberstein, Germany) with angled holes, as presented in the CAD design in Figure S1a from the Supplementary Information (SI), was utilized. Due to the angled holes (74° from backside to frontside), cracks in the diamond film should be prevented, since overetching towards the membrane edges is minimized (for further details see [46]). The mask features distinct hole sizes, so that membranes with different diameters can be created (see Figure S1b). 

As illustrated in Figure 1 (4), the sample setup in the ICP-RIE consisted of the SCD plate with the diamond bulk mask on top of it mounted on a quartz glass substrate. The diamond bulk mask was just pressed with a tweezer on top of the sample. The membrane structures were prepared in the ICP-RIE system similar to the strain relief etch with alternating Ar/Cl_2_ and O_2_ steps. Additionally, the “soft” etch recipe can be repeated to further decrease the roughness of the fabricated membrane structures (Figure 1 (5)). Some details regarding etch time/etch rate and recipe alterations for a further reduction in surface roughness are elucidated in Section 3.1.3. For the following characterization in a fiber-based microcavity (Figure 1 (6)), the adhesive layer was removed, the cleaning process was repeated as explained before and the sample was bonded via van der Waals forces to the respective cavity mirror.

### 2.2. Morphological Characterization and Surface Analysis of SCD Samples

Besides an optical evaluation with a white light interferometer (WLI, Zygo New View 5000, Zygo Corporation, Middlefield, CT, USA), the surface topography was investigated using the tapping mode of an atomic force microscope (AFM, DualScope 95, DME, Semilab Germany GmbH, Braunschweig, Germany). The derived data were processed and evaluated with the freeware *Gywddion Version 2.52* (2018). Additionally, overview images of the individual membrane structures were recorded with a confocal laser scanning microscope (Keyence VK-X1000, Keyence Deutschland GmbH, Neu-Isenburg, Germany) and of the diamond bulk mask with a light microscope (Leica DMR Microscope, Leica Microsystems, Wetzlar, Germany). To gain more knowledge about the thickness variation along the sample and of the created membrane thickness, again the same WLI was utilized as well as a profilometer (Ambios XP-100, Santa Cruz, CA, USA). The surface composition of the SCD samples was investigated using X-ray photoelectron spectroscopy (XPS) applying an Axis Nova Spectrometer (Kratos Analytical Ltd., Manchester, UK) with monochromatic Al Kα radiation. For the acquisition of the survey spectra, the transmission energy was set at 160 eV and the core spectra at 20 eV with an analyzed area of 400 × 700 μm^2^. The occurring surface charges were neutralized using a heated filament within a magnetic lens system (*I =* 1.9 A, *V* = 3.2 V). Elemental concentrations were calculated according to standard sensitivity factors supplied by the instrument’s manufacturer.

### 2.3. Bonding of SCD Membranes on Dielectric Mirrors and Cavity Characterization

Incorporating a diamond membrane in an optical cavity needs to be carried out in such a way that losses, e.g., due to scattering, are minimized. Therefore, we used van der Waals forces to bond the membrane onto a planar superpolished cavity mirror [47]. This technique is advantageous as very strong bonding forces between the sample and the dielectric mirror coating lead to negligible air gaps between the surfaces. This is crucial because an additional air gap would introduce another diamond–air interface and thus increase the scattering losses in the cavity system. As van der Waals forces between two flat surfaces attenuate with the distance cubed [48], the surfaces have to be very clean without any residual particles in between.

**Figure 2:**
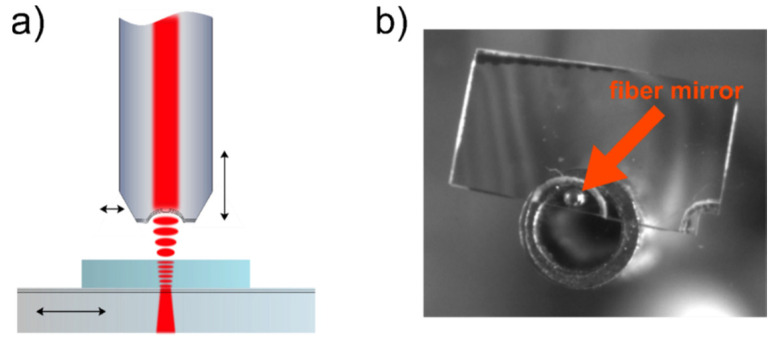
A tunable fiber-based microcavity: (**a**) schematic drawing of the system. The mirror can be moved laterally for coarse positioning while the fiber can be precision-positioned both laterally and longitudinally; (**b**) microscopic image of the cavity, taken from behind the plane mirror with a CMOS camera. One can see the fiber which is glued onto a needle for stability and the bonded diamond sample. Interference fringes that appeared after bonding the sample are visible.

In order to characterize the bond quality as well as the optical properties of the diamond membranes, we performed cavity-based measurements in a homebuilt fiber-based Fabry–Pérot scanning microcavity setup [49]. Such a cavity setup features a high tunability regarding both the cavity mirror distance, which controls the resonance frequency, as well as the lateral position of the cavity on the sample to probe the topology of the membrane (Figure 2a). The setup consists of a planar mirror on which the diamond sample is bonded and a concave micromirror which is produced on the end facet of a single-mode optical fiber using a pulsed CO_2_ laser [30] and subsequently coated with a distributed Bragg reflector (DBR) coating. The fiber therefore acts both as a cavity mirror as well as an efficient way of coupling light into and out of the cavity. 

The planar mirror can be moved laterally for coarse alignment. For precise lateral alignment, the fiber can be raster-scanned and positioned using piezo-electric transducers. Another piezo was used to move the fiber perpendicular to the mirror surface to scan the mirror separation. To probe the cavity, a narrow-band laser was coupled into the cavity fiber and the transmitted light was detected by an avalanche photodiode (APD). Figure 2b shows a camera image taken from behind the cavity mirror. Our cavity consists of a single-mode optical fiber mirror with a transmission of *T^f^* = 52 ppm at 637 nm (as measured by the manufacturer) and a radius of curvature of ~30 µm as measured with a home-built WLI. The coating on the macroscopic cavity mirror is specified for a transmission of 57 ppm at 637 nm leading to an expected maximum finesse for the bare cavity of ~57,600. We coupled light of a tunable, narrow-linewidth laser (<1 MHz) operating around 639.7 nm into the cavity and recorded the transmission with an APD, while periodically scanning the cavity length at 10 Hz using a piezo actuator.

## 3. Results and Discussion

### 3.1. Thin Single-Crystal Diamond Membranes

In the following, the optimizations and results regarding the structuring process of SCD membranes are described referring to the individual fabrication steps. 

#### 3.1.1. Cleaning Procedure

To yield good quality membranes, it is of utmost importance to start with a clean surface since small particles and impurities arising from polishing can induce micromasking (e.g., nanopillar and hole formations) in the following dry etching procedures. Besides solvent cleaning, a cleaning process with piranha solution was included to remove organic contaminations. Furthermore, the samples can be cleansed, while mounted on quartz glass, since the WaferBOND^®^ adhesive layer is not corroded by piranha solution. 

**Figure 3:**
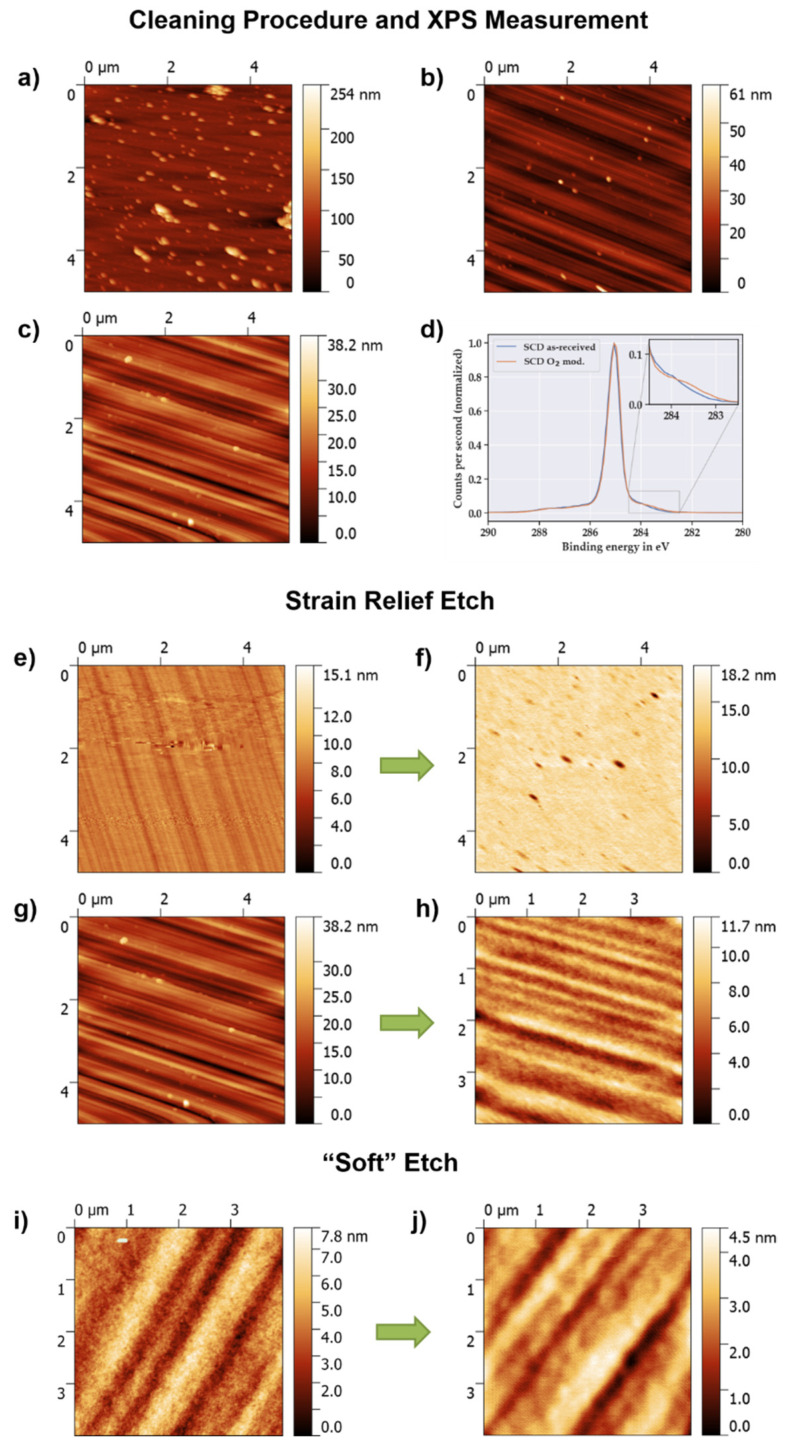
Atomic force microscopy (AFM) images of SCD surfaces after cleaning, XPS C1s core spectra and in comparison before and after strain relief etch and “soft” etch: (**a**) after first cleaning procedure (rms ~ 20 nm) and (**b**) after second cleaning procedure with piranha solution (rms ~ 4.4 nm); (**c**) after oxygen plasma asher treatment (rms ~ 4 nm); (**d**) XPS C1s core spectra measured for general grade SCD sample as-received from the manufacturer and after treatment in the oxygen plasma asher, with an inset showing the lower binding energy site. (**e**) General grade SCD with an initial low surface roughness (rms ~ 0.6 nm) before and (**f**) after strain relief etch (50 min Ar/Cl_2_ + 90 min O_2_), including some hole formation due to micromasking and a similar roughness without considering the holes (rms ~ 0.7 nm); (**g**) electronic grade SCD exhibiting distinct polishing grooves (rms ~ 4 nm) before and (**h**) after strain relief etch with a cyclic Ar/Cl_2_ + O_2_ recipe, which leads to an improved smoothening of the surface (rms ~ 1.6 nm); (**i**) electronic grade SCD surface before (rms ~ 1.2 nm) and (**j**) after (rms ~ 0.76 nm) a “soft” etching with Ar/Cl_2_ (40 min). The white line in the upper left corner of (**i**) depicts an AFM artifact, which was not considered in the height plot and hence in the surface roughness calculation.

As shown in the AFM evaluation in Figure 3a–c for an electronic grade SCD sample, which exhibited a notable amount of surface impurities to begin with, several cleaning processes were needed. After a 45 min long piranha solution bath, the surface still featured a significant number of particles with heights of up to 250 nm (Figure 3a), which led to high root mean square (rms) surface roughness values in the range of 20 nm. However, with a repetition of the acidic cleaning for 1 h, the amount of particles was drastically reduced leading to lower rms values of 4.4 nm (Figure 3b). The notable grooves and striations observed in the AFM images are typical defects caused by mechanical polishing [41].

Additionally to the cleaning with piranha solution, the sample was cleansed and O-terminated in an oxygen plasma asher with a relatively low power of 150 W so that the surface was not significantly etched (Figure 3c). Besides a reduction in height and number of particles, metal impurities at the surface can be removed. The surface compositions of a general grade SCD sample as-received from the manufacturer, without any cleaning, and a general grade SCD plate after oxygen treatment in a plasma asher (SCD O_2_ mod.) was investigated by XPS. The results for the elemental surface composition are summarized in Table 1, while the C1s core spectra of both samples are depicted in Figure 3d. The spectra are set to a binding energy of 285.0 eV to compensate for energy shifts due to charging effects under the influence of the neutralizer.

According to the elemental surface composition, the as-received SCD sample exhibited metal impurities from cobalt and zinc (0.1 at%), which might be incorporated during the polishing procedure, as well as nitrogen (0.56 at%) and oxygen (6.6 at%) due to surface contaminations of the sample. These impurities can be removed by treatment with oxygen plasma as demonstrated for the SCD O_2_ mod. sample, which did not show any detectable content of cobalt, zinc and nitrogen at the surface. Furthermore, as revealed by the carbon core spectra (C1s), the peak shape of the as-received sample is rather similar to that of the SCD O_2_ mod. sample. In particular, regarding the lower binding energy site (283–284 eV), no notable sp^2^ carbon content, which might by formed by graphitization and/or amorphization due to ion bombardment by the plasma treatment, seemed to be present [50] (see Figure 3d inset). Otherwise, a more prominent peak shoulder at ca. 284 eV would be observed.

#### 3.1.2. Optimizations in the Surface Roughness

Since most of the commercially purchased SCD plates are double-side polished, they can possess polishing damage induced dislocations and defects as well from both sides. Therefore, to reduce the stress in the diamond sample caused by this, a strain relief etch of several microns from its backside was executed. A smooth and defect-reduced surface of the sample backside can be an advantage—e.g., for the later incorporation of color centers. A high surface roughness caused by this highly strained layer can lead to spin decoherence and an unstable charge state of NV centers [51]. As reported by Sangtawesin et al. [52], rougher surface morphologies can increase the number of unoccupied defect states with unpaired electrons, which can act as electronic traps and hence induce both magnetic and electric field noise leading to NV spin decoherence. Additionally, a more planar surface from the backside could lead to an improved bonding quality on the dielectric mirror for cavity experiments. 

Quartz glass was utilized as a substrate and carrier chip to avoid micromasking since it is not severely etched during the following Ar/Cl_2_ + O_2_ procedure. Otherwise, sputtered and redeposited material could lead to hole formation on the diamond surface as would be the case for, e.g., a silicon substrate or a carrier chip. Additionally, based on AFM evaluations, Ruf et al. described in more detail how an etching process with only O_2_ can lead to the formation of etch pits due to the micromasking effect [24]. Small diamond particles, introduced by, e.g., the polishing procedure, were firstly etched with a lower etch rate to nanopillars with trenches around them. These trenches widened with longer etching procedures, while the nanopillars were eventually etched away. Such pits can lead to NV spin decoherence due to an alteration in the electron structure of the diamond surface in comparison to smoother surfaces without such defects. As derived from the XPS measurements of Sangtawesin et al. [52], the C1s core spectrum from such a rough surface with pits features a more prominent sp^2^ carbon peak leading to this different electronic characteristic. Therefore, it is of importance to maintain or generate a planar surface to yield good quality SCD membranes. 

With the Ar/Cl_2_ step, diamond particles that originate from the polishing procedure or sputtered material due to etching and that could cause micromasking are removed. Besides its function as a cleaning step, it provides a smoothing effect due to an isotropic etching of diamond [53]. By sputtering with Ar, the surface is activated, so that carbon atoms react with chlorine to the volatile etch product CCl_x_ [54]. Afterwards, the O_2_ step is utilized for structuring and hence deep-etching into the diamond with a high etch rate (~60 nm min^−1^). 

For a strain relief etch, about 7 µm were removed from the backside of a general grade SCD plate by the application of 50 min Ar/Cl_2_ and 90 min O_2_. As evaluated from the left AFM image presented in Figure 3e, the initial surface roughness of the sample was in a lower range with rms values of 0.6 nm over a 5 × 5 µm^2^ area and it possessed a lower extent of notable polishing grooves. It should be noted that the horizontal lines are artefacts from the AFM measurement and do not represent the actual surface topography. After removal of several microns (Figure 3f), the overall max. height and surface roughness were in a similar range as before etching (rms ~ 0.7 nm, without consideration of the pits). Even though micromasking was reduced compared to other results in the literature [42,52,55], where only oxygen-based etching was applied, small holes were still present. In particular, the improvement in the surface roughness is less effective if the initial roughness is in the range of several nanometers due to polishing damages (cf. Figure 3c). Therefore, the extent of polishing defects is not reduced and hence the quality of the membranes structured afterwards is not sufficient for cavity experiments due to increased losses. 

Hence, it seems that one longer cleaning/smoothening step with Ar/Cl_2_ at the beginning is not sufficient for minimizing micromasking and an enhanced planarization of the surface. Therefore, an alteration of the recipe was applied. Besides one long Ar/Cl_2_ step at the beginning to remove particles from the surface, 5 min Ar/Cl_2_ steps (at the same substrate temperature as for the O_2_ steps) in between 15 min long O_2_ steps were applied. These short Ar/Cl_2_ steps removed particles sputtered during oxygen etching more efficiently to minimize the micromasking and, additionally, promoted the smoothing effect. With this cyclic recipe, a strain relief etch was executed for an electronic grade SCD sample with a rougher surface (rms ~ 4 nm) due to deep polishing grooves and particles (Figure 3g). With the same total etch time of 90 min for the O_2_ steps, this cyclic recipe led to a significant reduction in the polishing grooves and of the surface roughness (rms ~ 1.6 nm) as presented in the AFM evaluation in Figure 3h. Besides an enhanced smoothening of the surface, more than halving the surface roughness, it is evident that due to the repeated Ar/Cl_2_ steps micromasking and hence hole formation were minimized. 

Nevertheless, we aimed for a further planarization of SCD surfaces with higher roughnesses (above 1 nm on the same area scale). Even after several etch processes with the cyclic Ar/Cl_2_ + O_2_ recipe, the profile of the polishing grooves was just transferred by etching into the diamond maintaining the surface roughness. For this, an altered Ar/Cl_2_ etch recipe with a low DC bias value and a low etch rate of a few nanometers per minute was utilized. With a low or near zero bias value, the acceleration of the etching species towards the SCD surface is lowered or even prevented. Therefore, the physical sputtering part of the etching mixture is reduced, while isotropic chemical reactions become more dominant. As described in more detail by Radtke et al. [45], it is assumed that by this enhanced chemical etching the reduced amount of etching species bombardment leads to less damage to the SCD surface and hence to a further improvement in the surface roughness.

In Figure 3i,j, AFM images of an electronic grade SCD surface before and after the application of 40 min “soft” etch Ar/Cl_2_ are presented. With the “soft” etch, the rms surface roughness decreased from 1.2 nm over a 4 × 4 μm^2^ area to 0.76 nm, also including a reduction in the groove’s maximum height. The problem seems to be that deeper grooves and defects are still just transferred by the etching procedure instead of being removed. Hence, more systematic etching experiments needs to be conducted or other planarization methods need to be applied such as noncontact polishing [56] or high-precision mechanical polishing [57] to remove the defects completely and to obtain atomically flat diamond surfaces.

#### 3.1.3. Structuring of SCD Membranes 

To increase light-matter coupling in a cavity, cavities with small mirror separations are beneficial, such that the introduction of the thinnest possible membranes are beneficial. On the other hand, maintaining optical and spin coherence requires sufficient separation of color centers from interfaces, and it was found that a diamond thickness of 3–5 µm represents an optimal choice for NV centers [24]. Such thin structures become mechanically fragile, and one approach is to structure circular membranes in the diamond sample. By using the diamond bulk mask with angled holes (cf. Figure S1), different membrane diameters due to variant mask holes can be created via ICP-RIE. In the following, membranes generated with the two largest mask apertures will be presented, since these membranes provided a sufficient diameter for operation with the cavity fiber and showed, in general, a more homogenous thickness in comparison to the smaller structured membranes.

**Figure 4:**
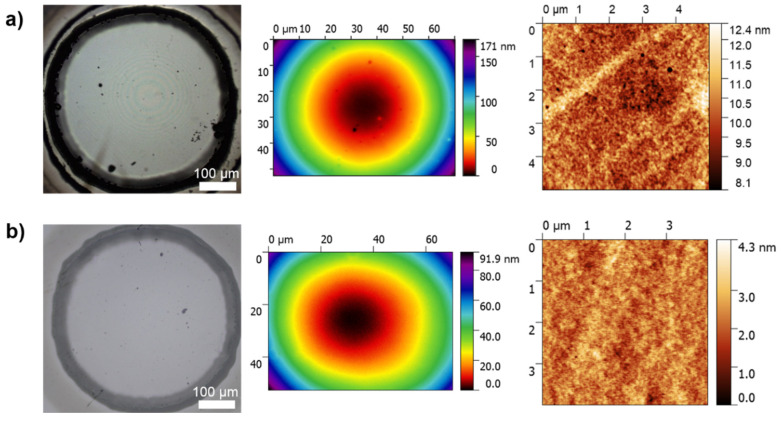
Comparison of the morphological characterization of SCD membranes, from left to the right: overview via optical micrograph, white light interferometer (WLI) measurement from the membrane center, AFM image from the center: (**a**) general grade SCD membrane (∅ = 470 μm, thickness 2–3 μm, rms ~ 0.7 nm over a 5 × 5 μm^2^ area) structured with one long Ar/Cl_2_ cleaning step and one long O_2_ structuring step (in total three etch processes); (**b**) general grade SCD membrane (∅ = 510 μm, thickness 5 μm, rms ~ 0.4 nm over a 4 × 4 μm^2^ area) structured with cyclic Ar/Cl_2_ + O_2_ recipe (in total three etch processes).

For etching SCD membranes, the same criteria are valid as outlined for the strain relief etch—i.e., for yielding good quality membranes, the micromasking effect and defects originating from the polishing procedure need to be minimized. Therefore, the membranes were structured with alternating Ar/Cl_2_ + O_2_ etching steps. In Figure 4, morphological characterization results of SCD membranes from two different samples are illustrated. These general grade SCD membranes were fabricated with variations in the etching recipe. It should be noted that both samples exhibited a similar initial rms surface roughness of 0.6–0.7 nm over a 5 × 5 μm^2^ and 4 × 4 μm^2^ area, showing a lesser extent of polishing damages as outlined, e.g., for the electronic grade SCD sample in Figure 3g. Details about the exact etching durations and mask diameters are given in the Supplementary Information.

The first presented membrane (Figure 4a) with a thickness of 2–3 μm and a diameter of 470 μm was structured via one long Ar/Cl_2_ cleaning step and one long O_2_ structuring step. These results with only one longer cleaning step in the beginning correlate with the observations made in Section 3.1.2. It depicts the lacking improvement of surface roughness (rms ~ 0.7 nm, third image in Figure 4a) and the presence of some holes due to micromasking, seen as black dots in the optical micrograph as well as in the WLI measurement of the membrane center, showing the thickness variation along a larger area.

A notable improvement in the overall quality can be recognized by the second presented membrane (Figure 4b), with a thickness of ca. 5 μm and a diameter of 510 μm, which was structured by the cyclic Ar/Cl_2_ + O_2_ recipe. Etching with short Ar/Cl_2_ steps in between the O_2_ steps provides a more efficient removal of particles and improvement in smoothening. As a result, the micromasking effect was significantly decreased as it can be seen by the reduced number of holes (black dots) in the optical microscope image and WLI measurement of the membrane center. In particular, with a reduced number of defects the surface roughness improved, exhibiting a planar surface with an rms value of around 0.4 nm. As outlined in the introduction, this surface roughness value is sufficient for an incorporation into a fiber-based microcavity system to achieve high finesse and prevent losses. 

### 3.2. Van der Waals Bonding Procedure 

**Figure 5:**
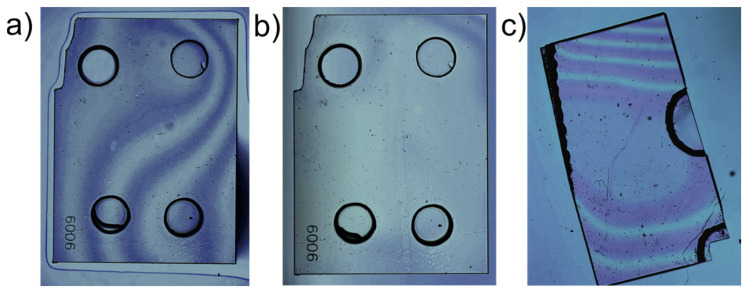
Microscopic images showing the bonding process: (**a**) the electronic grade SCD before and (**b**) after the bonding process. Before the bonding process has finished, one can clearly see the residual water around the sample edges as well as the interference fringes that occur due to the gap between both surfaces; (**c**) microscopic image of the processed general grade SCD sample after the bond. As the sample was broken in half during the cleaning process, the membrane is now located on the edge of the sample.

In the following, we describe the procedure that was successfully used to bond several samples on plane mirrors with dielectric coatings that were specified by the manufacturer to have an rms surface roughness of <0.2 nm. The samples and the mirrors were cleaned in a cleanroom environment using piranha solution (2:1 H_2_SO_4_ [98%]:H_2_O_2_ [35%]; for details, see Supplementary Information). A sketch of the respective steps is presented in Figure S2. To achieve bonding of the diamond sample on a mirror, the two surfaces need to be close enough together so that the van der Waals forces are strong enough to build the bond. Therefore, we make use of the capillary effect from a thin water film in between. Previous bonding attempts indicate that only a very small amount of water is needed to pull the sample close enough to the mirror surface. Hence, we do not add any additional water for the bonding process but instead pick up the sample directly from the water surface that is used for the last cleaning step and utilize the residual water to mediate the bond. Due to the surface activation of the mirror, the water droplets under the sample automatically distribute homogeneously over the bonding region. Placing the mirror under a light microscope with bright illumination allows the observation of the bonding procedure while accelerating the process as it can be seen in Figure 5.

If both surfaces are clean enough, interference fringes will become visible after placing the sample on the mirror, as seen in Figure 5a. As the water evaporates, the interference fringes will expand, which indicates a smaller gap between the diamond and the mirror coating. When all the residual water has evaporated and there are only few fringes left, the bonding process is finished (Figure 5b). All successful approaches have shown that this only takes a few minutes. Afterwards, the bond quality can be checked by gently pushing the sample with a pair of tweezers. A successful van der Waals bond should be very strong and therefore leave the diamond motionless under lateral push. Figure 5c shows the bonding result of the general grade SCD sample which has been used for most of the cavity characterization measurements. The interference fringes indicate a very good bond quality in the center of the sample as well as in the thinned down membrane, while the outer region shows an increasing angle between the diamond sample and the mirror surface.

### 3.3. Characterization of SCD Membranes in a Fiber-Based Microcavity

**Figure 6:**
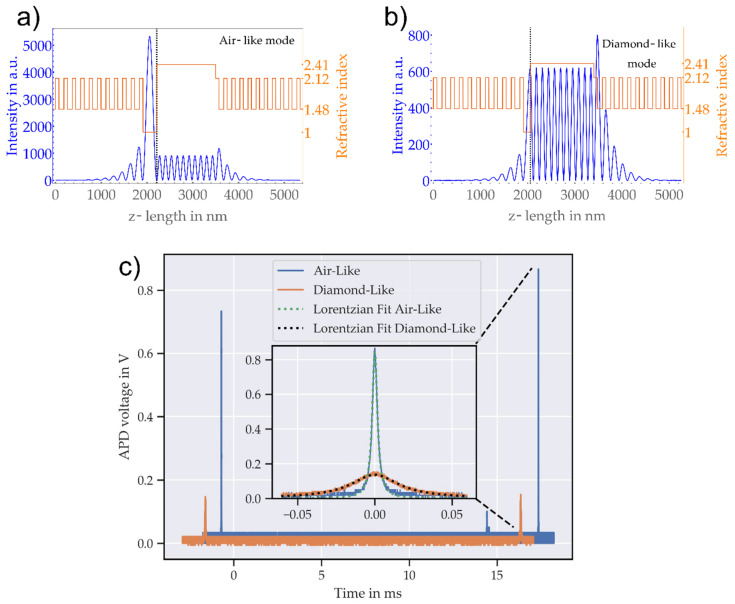
Numerical simulations of a 1D model of the cavity and related finesse measurements: (**a**) The electrical field intensity of a hybrid cavity system (blue). The orange lines show the refractive indices in the respective areas (from left to right: fiber mirror, air gap, diamond membrane, planar mirror); (**b**) the same simulation for a different diamond thickness, leading to a diamond-like mode. Both simulations are performed with the same mirror transmission without diamond which leads to a higher field in the air-like case. This occurs because the diamond membrane alters the transmission of the planar mirror, depending on the diamond thickness; (**c**) longitudinal cavity modes for determining the cavity finesse: a narrow linewidth laser, running at a wavelength of 639.7 nm, is coupled in the cavity and the length is scanned periodically. Blue and orange curves show measured voltage over oscilloscope time base for two different positions on the membrane. We attribute the two positions to diamond-like (orange) and air-like (blue) modes. The inset shows a centered zoom into the air-like and the diamond-like fundamental modes. Fits to the data reveal the expected Lorentzian shape of cavity modes. The FWHM at the diamond-like position on the sample is much larger than at the air-like position, yielding a much lower finesse.

Due to the different refractive indices of air and diamond ($n_{d}\approx 2.41$), Ref. [58] there is an additional partly reflecting interface introduced to the cavity system which leads to mode hybridization [59]. Depending on the thickness of the diamond membrane, the field distribution inside the cavity will change. We will focus on two special cases, henceforth referred to as “diamond-like” and “air-like” (Figure 6). The “air-like” case will occur for a diamond thickness of $m\cdot \lambda /2n_{d}$. In this case, the electric field will have a node at the diamond–air interface (Figure 6a). If the diamond membrane thickness equals $\left(m+1/2\right)\cdot \lambda /2n_{d}$ with m being an integer, the electric field will have an antinode at the diamond–air interface (Figure 6b) and the field intensity in the membrane will be equal to the intensity in the air gap. The cavity is then less susceptible to scattering losses due to roughness on the diamond surface, but the majority of the field intensity is located in the cavity air gap. Both simulations are performed with the same mirror transmission without diamond which leads to a higher field in the air-like case. This occurs because the diamond membrane alters the transmission of the planar mirror, depending on the diamond thickness. Therefore, the intensity units are arbitrary and not comparable for both cases. Since a high electric field is beneficial for most light-matter-interaction applications, such as coherent coupling to NV centers, an operation on a diamond-like mode is desirable. Thus, it is crucial to characterize the performance of the membrane-cavity system with respect to its losses, which will directly affect the finesse and thereby the Purcell enhancement.

We performed finesse measurements at several locations on the membrane by scanning the mirror separation and probing the cavity transmission. The scanning range was set to include more than one free spectral range (FSR), as shown in Figure 6c. We measured the finesse *F* by fitting Lorentzian curves to two neighboring fundamental modes and calculating the finesse via F=FSR/FWHM, where FWHM is the full width at half maximum of the resonance. We note that this measurement technique is affected by piezo nonlinearities and introduces an uncertainty of about 10% for the used measurement settings.

**Figure 7:**
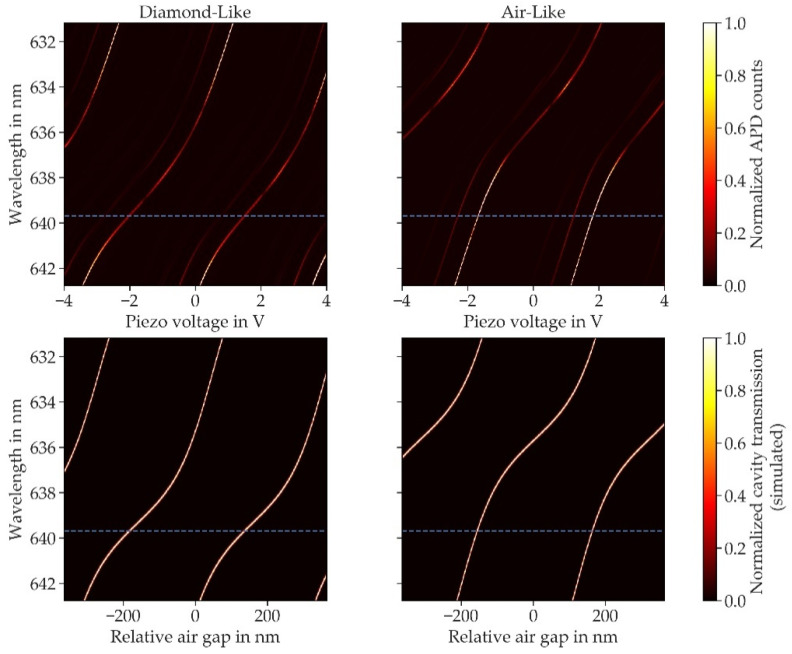
Hybridized mode structure at different positions on the membrane: the upper panel shows the measured mode dispersion on two different positions. The blue dashed line indicates the wavelength of the laser used for finesse measurements. The first position (left panels) is very close to a diamond-like mode whereas the second position (right panels) approaches an air-like mode. The lower panels show transmission simulations using a matrix transfer method. Diamond thicknesses of 5.995 (air-like) and 5.900 µm (diamond-like) yield the best agreement with the measured data.

With this, we found finesse values within a range of 500 up to 5000 for different positions on the membrane. To correlate the change in finesse with the mode structure, we measured the mode dispersion on two distinct positions on the sample. Therefore, we coupled light from a supercontinuum laser source into the cavity and measured the transmission spectrum with a spectrometer (*Andor Shamrock 500i*) as a function of the cavity length. The cavity length was changed stepwise by a piezo, and a spectrum was taken for each cavity length, revealing the dispersion of the cavity modes. The wavelength calibration of the spectrometer was carried out using light from a Ne-Ar lamp. For the first position, we found the mode structure at 637.9 nm to be very close to the flattest position of the dispersion, indicating a mode with a diamond-like character as shown in the upper panel of Figure 7.

The dispersion at the second position of the sample reveals a mode with an air-like character. Simulations of the mode dispersion using a transfer-matrix model and layer parameters of the mirror coatings as provided by the manufacturer agree well with our measurements (see lower panel of Figure 7). For the simulations, we used a diamond thickness of around 6 µm, in good agreement with WLI measurements of the membrane, and an air gap of around 4 µm and then fine-tuned the lengths to fit the measured mode structure.

For further investigation of the mode dependent losses, we measured the finesse on the two positions again with more than 200 measurements each for gathering sufficient statistics. The measurements were carried out directly after the respective dispersion measurement to minimize possible drifts of the lateral position. We found a finesse of $F_{a}=\;$5252 ± 230 for the air-like mode and the lowest value of $F_{d}=$ 555 ± 5, which we linked to the diamond-like mode. While the finesse in air-like regions is mostly constant, it shows large variations for the diamond-like mode, with finesse values of up to 2000. Using again the transfer-matrix model, we simulated the transmission of the planar cavity mirror for the two diamond thicknesses, including an additional absorption loss of 10 ppm, which leads to mirror losses of $L_{a}^{m}$ = 83 ppm and $L_{d}^{m}$ = 344 ppm. Taking into account the mode-dependent weighting of losses on the fiber mirror side, we estimated those losses for the air-like mode using [39]

$$n_{d}\cdot L^{f}=\frac{2\pi }{F_{a}}-L_{a}^{m}$$

to be $L^{f}=\;$462 ppm to fit the measured finesse, with the refractive index of diamond $n_{d}=2.41$. This loss is larger than expected from the mirror coating, but similar values for the specific cavity used here are consistently found also for measurements without diamond in the cavity under otherwise identical conditions. We thus ascribed the extra loss to scattering at the fiber mirror due to misalignment or defects on the fiber. For the case of the diamond-like mode, we expected additional losses due to scattering on the diamond–air interface as well as the increased transmission on the mirror side. Using [39]

$$L_{d}^{ad}=\frac{2\pi }{F_{d}}-L_{d}^{m}-\frac{L^{f}}{n_{d}}$$

with the fiber losses found above and the simulated mirror transmission, we estimated additional losses of $L_{d}^{ad}=$ 2000–11,000 ppm to fit the measured finesse of the diamond-like mode. Contributing these additional losses mainly to scattering at the diamond–air interface for a pure diamond-like mode [39]

$$L_{d}^{S}=\frac{\left(1+n_{d}\right)*{\left(1-n_{d}\right)}^{2}}{n_{d}}{\left(\frac{4\pi \cdot \sigma_{d}}{\lambda }\right)}^{2}$$

would correspond to an rms roughness of $\sigma_{d}$ = 1.35–3.15 nm. This is much larger than the measured value of <0.5 nm on the specific surface area of the cavity measurements, such that a dominant contribution to the loss of about 10,000 ppm remains unexplained. We ruled out the possibility that local surface defects or contamination are the origin by probing several locations on the diamond membrane. We note that our calculations are based on pure air- and diamond-like modes, whereas our dispersion measurement has shown slight deviations from the pure cases at our measurement positions. Such deviations lead to even lower expected losses for a given roughness. We thus need to conclude that the model used to explain the loss neglects a significant additional contribution, and further work is required to analyze its origin.

## 4. Conclusions

With the presented ICP-RIE approach, which combines alternating Ar/Cl_2_ + O_2_ etching steps and the application of a diamond bulk mask, including angled holes, thin SCD membranes, with thicknesses between 2 and 5 μm and varying diameters, were fabricated. The quality of the surface was improved by the application of one long Ar/Cl_2_ cleaning step at the beginning and short Ar/Cl_2_ steps between O_2_ structuring steps with a high etch rate for deep etching into the diamond. This reduced the micromasking and hence the further defect formation during the etching. Additionally, the smoothening effect of the isotropic Ar/Cl_2_ steps was exploited to planarize the surface and reduce the distinct polishing grooves and striations—particularly for rougher samples (rms roughness values of several nms) with the “soft” etch Ar/Cl_2_ recipe, applying a low DC bias value (~40 V). With the optimized recipe, SCD membranes with an rms roughness value down to 0.4 nm over a 4 × 4 μm^2^ area and without a significant amount of micromasking induced pits were fabricated.

The membranes were successfully integrated into a fiber-based Fabry–Pérot microcavity by van der Waals bonding to a plane cavity mirror. We have measured and modelled the mode structure that occurs due to the coupling of a diamond membrane to a cavity and determined the losses for air-like and diamond-like modes. Operation under air-like modes did not introduce additional loss, while for a diamond-like mode we observed a significant additional loss which cannot be explained by surface roughness only and requires further investigation. The achieved parameters of the integrated system can enable an up to 10-fold enhancement of the ZPL emission of NV centers and are thus promising for efficient spin readout and quantum network applications. 

## Figures and Tables

**Table 1:** Elemental surface composition of SCD general grade samples as-received and after oxygen plasma asher treatment as determined by X-ray photoelectron spectroscopy (XPS).

Type	C [at%]	O [at%]	F [at%]	Co [at%]	Zn [at%]	N [at%]
single-crystal diamond (SCD) as-received	92.5	6.6	0.1	0.1	0.1	0.56
SCD O_2_ mod.	94.7	5.0	0.2	0.03	0.02	0.01

## Supplementary Materials

The following are available online at https://www.mdpi.com/2072-666X/11/12/1080/s1, Figure S1: Geometry of the used diamond bulk mask with angled holes and the sample setup in the ICP-RIE chamber, Figure S2: Schematic of the bonding sequence.
