# Supplementary material for: Fabrication and Characterization of Single-Crystal Diamond Membranes for Quantum Photonics with Tunable Microcavities
Source: Micromachines (Basel). 2020 Dec 4;11(12):1080. doi: 10.3390/mi11121080 (PMC7762039; doi:10.3390/mi11121080)
Supplement: Supplementary file 1 [file micromachines-11-01080-s001.pdf]

micromachines

Supplementary Information: Fabrication and Characterization of Single-Crystal Diamond Membranes for Quantum Photonics with Tunable Microcavities

Julia Heupel [1,†], Maximilian Pallmann [2,†], Jonathan Körber [2,†], Rolf Merz [3], Michael Kopnarski [3], Rainer Stöhr [4], Johann Peter Reithmaier [1], David Hunger [2,5] and Cyril Popov [1,*]

1 Institute of Nanostructure Technologies and Analytics (INA), Center for Interdisciplinary Nanostructure Science and Technology (CINSaT), University of Kassel, Heinrich-Plett-Straße 40, 34132 Kassel, Germany; j.heupel@ina.uni-kassel.de (J.H.); reithmaier@ina.uni-kassel.de (J.P.R.)
2 Physikalisches Institut, Karlsruher Institute für Technologie (KIT), Wolfgang-Gaede-Str.1, 76131 Karlsruhe, Germany; maximilian.pallmann@kit.edu (M.P.), jonathan.koerber@student.kit.edu (J.K.); david.hunger@kit.edu (D.H.)
3 Institut für Oberflächen- und Schichtanalytik (IFOS), University of Kaiserslautern, Trippstadter Str. 120, 67663 Kaiserslautern, Germany; merz@ifos.uni-kl.de (R.M.); kopnarski@ifos.uni-kl.de (M.K.)
4 3. Physikalisches Institut, University of Stuttgart, Pfaffenwaldring 57, 70569 Stuttgart, Germany; rainer.stoehr@pi3.uni-stuttgart.de
5 Institut für QuantenMaterialien und Technologien (IQMT), Karlsruher Institut für Technologie, Hermann-von-Helmholtz-Platz 1, 76344 Eggenstein-Leopoldshafen, Germany
* Correspondence: popov@ina.uni-kassel.de; Tel.: +49-561-804-4205
† Authors contributed equally to this work.

Received: 11 November 2020; Accepted: 02 December 2020; Published: date

Design of the diamond bulk mask for membrane fabrication

Due to the long etching procedures to remove more than 20 µm of SCD, a diamond bulk mask was utilized as a resistive etch mask. As outlined in Figure S1a the mask included angled holes (74 ° from backside to frontside) with varying inlet diameters to create membranes with distinct sizes (cf. optical microscope images in Figure S1b). With these angled mask holes overetching towards the diamond membrane edges should be prevented in comparison to straight mask holes.

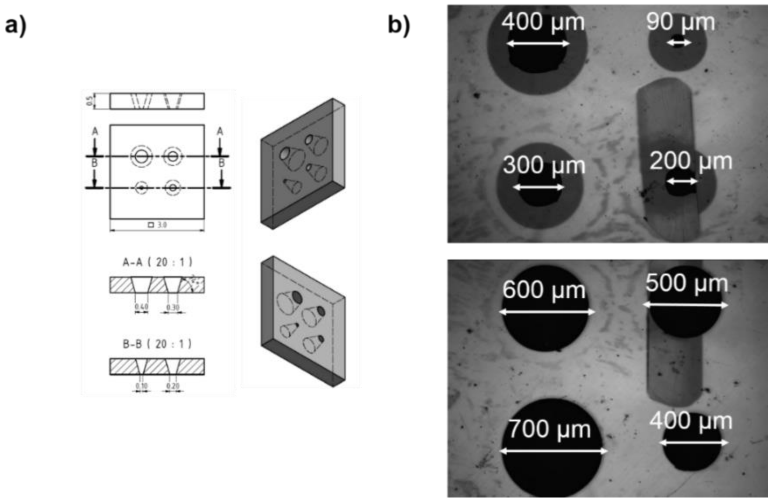

Figure S1. Geometry of the used diamond bulk mask with angled holes and the sample setup in the ICP-RIE chamber: (**a**) Technical drawings of the bulk diamond mask with angled holes; (**b**) Optical microscope images depicting the dimensions of the mask inlets from the frontside (upper image) and from the backside (lower image).

Technical details about the membrane fabrication

For the first presented membrane (cf. Figure 4a) a thickness of 2-3 µm was achieved by the removal of ca. 21 µm via one long Ar/Cl_2 cleaning step and one long O_2 structuring step, including in total three etch processes (1st etch: 50 min Ar/Cl_2 + 90 min O_2, 2nd etch: 60 min Ar/Cl_2 + 120 min O_2, 3rd etch: 60 min Ar/Cl_2 + 100 min O_2). A diameter of 470 µm was structured by etching through the second largest inlet in the diamond bulk mask (∅ = 300 µm from the frontside, ∅ = 600 µm from the backside).

The second presented membrane (cf. Figure 4b) with a thickness of ca. 5 µm was etched through the largest inlet in the diamond bulk mask (∅ = 400 µm from the frontside, ∅ = 700 µm from the backside). For the removal of ca. 22 µm from the diamond plate the cyclic Ar/Cl_2 + O_2 recipe with in total three etch processes was applied. Each etch processes consisted out of one long 50 min Ar/Cl_2 cleaning step and then six times alternating 5 min Ar/Cl_2 and 15 min O_2 steps, for the first etching procedure, eight times for the second and seven times for the last etch process.

Van der Waals bonding procedure

In the following, we describe the procedure that was successfully used to bond several samples on plane mirrors with dielectric coatings that were specified by the manufacturer to have a rms surface roughness of < 0.2 nm. A sketch of the respective steps is presented in Figure S2.

The samples and the mirrors were cleaned in a cleanroom environment using piranha solution (2:1 H_2SO_4 [98%]:H_2O_2 [35%]). For the mirror cleaning, the solution was prepared in a beaker. As soon as both acids start to react, the mirror was placed inside the beaker sitting on a heating plate, set to 90°C, to keep the mixture reactive throughout the cleaning process. After 20-30 minutes, the mirror was rinsed with double-distilled water several times to remove residual piranha solution Afterwards, the mirror was treated in an oxygen plasma (5 minutes at 150 W, oxygen flow of 25 sccm) to remove further organic contamination and to activate the mirror surface, leaving it hydrophilic.

The samples were cleaned in a similar way in piranha solution, but due to the smaller dimensions and the diamond brittleness, a careful handling is much more crucial. The sample was placed inside a filter crucible, inserted into a bigger beaker. Lifting and moving the sample was done using electronic vacuum tweezers (*POLYVAC-pickup, polyplas*) with a 0.8 mm inner diameter tip. After one hour in piranha solution at 90 °C, the filter crucible was moved into a clean beaker and rinsed with double-distilled water. This rinsing step was repeated several times until one can be sure that all residual acid has been washed away. At the last rinsing step, the filter crucible inside the beaker was filled with double-distilled water up to a very high level, such that the sample was swimming close to the edge of the vessel.

The sample can then be directly picked up from the water surface using the vacuum tweezers. Thereby, one makes sure that the bottom surface is not touching anything that might contaminate it and prevent a proper bond.

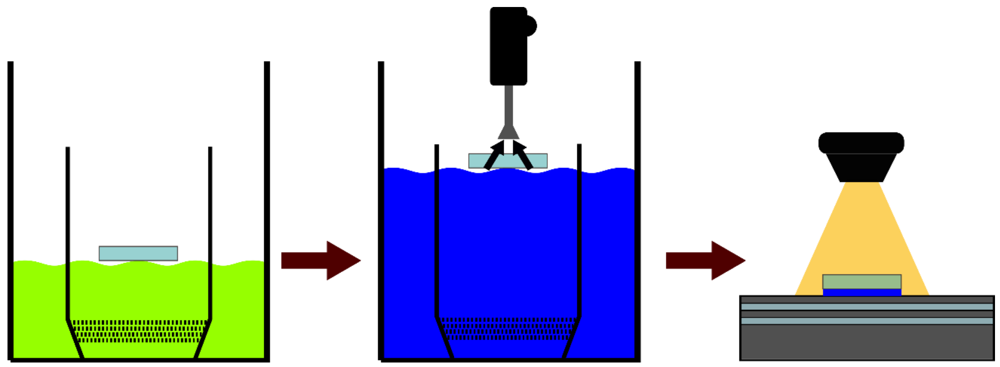

Figure S2. Schematic of the bonding sequence: The first step shows the cleaning in piranha solution. The sample swims on the surface, cleaning it from below where the bonding interface will be. In the second step, after cleaning, the beaker was filled with water to a high level, so one can pick up the sample from the water surface using the vacuum tweezers. The third step shows the observation and illumination under the microscope as well as the placement on the mirror.
